# Cerebrovascular dysfunction links aging to neurological disease

**DOI:** 10.18632/aging.103854

**Published:** 2020-07-29

**Authors:** Candice E. Van Skike, Veronica Galvan

**Affiliations:** 1 Barshop Institute for Longevity and Aging Studies and Glenn Biggs Institute for Alzheimer’s and Neurodegenerative Diseases, University of Texas Health, San Antonio, TX 78229, USA; 2 Department of Cellular and Integrative Physiology, University of Texas Health, San Antonio, TX 78229, USA; 3 Department of Veterans Affairs, South Texas Veterans Health Care System, San Antonio, TX 78229, USA

**Keywords:** mTOR, aging, Alzheimer’s disease, cerebral blood flow, cerebrovascular dysfunction

Aging is the greatest known risk factor for Alzheimer’s disease (AD). Brain aging is associated with structural and functional changes that increase the likelihood of developing a neurological disorder. Impaired cerebrovascular function, a universal feature of aging, is a biomarker for increased risk of AD, and is one of the earliest detectable changes in the pathogenesis of AD [1]. Indeed, chronic cerebral hypoperfusion typically develops nearly a decade prior to cognitive decline and precedes the presence of pathological hallmarks of AD, including brain atrophy and accumulation of β-amyloid and pathogenic tau [1]. In accordance with the two-hit vascular hypothesis of AD [2], these observations suggest that early age-associated cerebrovascular dysfunction may trigger the development of cerebrovascular pathology, driving cognitive impairment and accelerating the pathogenesis of neurological diseases of aging, including AD. Thus, cerebrovascular dysfunction may represent one of the earliest and most therapeutically addressable biological pathways underlying age-related cognitive impairment and neurological disease.

Research from our lab and others showed that the mechanistic/mammalian target of rapamycin (mTOR) drives several different aspects of cerebrovascular dysfunction in models of AD [3] and vascular cognitive impairment and dementia (VCID) [4], including blood-brain barrier (BBB) breakdown, cerebral hypoperfusion, reduced cerebrovascular reactivity, and impaired neurovascular coupling (please see [5] for a review of these mechanisms). We recently established that mTOR drives age-related cerebrovascular dysfunction in 34-month-old aged rats devoid of overt pathology or disease [6]. CBF deficits in aged rats were associated with microvascular rarefaction, synaptic loss, impaired neuronal network activation, and spatial learning and memory impairments. Chronic mTOR attenuation via rapamycin preserved cerebrovascular function and microvascular integrity, improved synaptic integrity and neuronal network activation throughout aging, and negated age-related cognitive decline in aged rats [6]. These recent results indicate that in addition to driving cognitive and cerebrovascular deficits in models of AD [3] and VCID [4], mTOR underlies the etiology of age-associated cerebrovascular and neuronal dysfunction during normative aging.

Since mTOR is a major regulator of aging [7], we propose that mTOR-dependent age-associated brain vascular dysfunction may be a central mechanism linking aging to the pathogenesis of AD and potentially other age-associated neurological diseases. Thus, mTOR may be a therapeutic target for age-related cerebrovascular dysfunction and cognitive decline that are not associated with specific disease processes. The negation of cerebral hypoperfusion and neurovascular uncoupling during normative aging by mTOR attenuation [6] suggests that inhibitors of the mTOR pathway could potentially target the earliest stages of preclinical AD to prevent further deterioration of cerebral perfusion, effectively decelerating progression to AD [1]. By restoring brain perfusion, mTOR attenuation would have the potential to delay disease onset or decrease severity of disease in AD, or mitigate age-related cognitive and cerebrovascular dysfunction in individuals that do not convert to AD. Therefore, clinical trials are warranted not only to test the ability of mTOR inhibitors to block or slow AD progression, but also to mitigate age-related cognitive decline.

In summary, mTOR is a common mediator of cerebrovascular dysfunction associated with multiple types of age-related cognitive impairment, including AD [3], VCID [4], and normative aging [6]. Thus, mTOR-driven cerebrovascular dysfunction may represent an early and possibly universal feature of aging that underlies cognitive impairment and the increased susceptibility of the elderly to specific forms of neurological disease, including but not limited to AD and related dementias.
